# Exercise-Induced Myokines With Therapeutic Potential for Muscle Wasting

**DOI:** 10.3389/fphys.2019.00287

**Published:** 2019-03-29

**Authors:** Rosanna Piccirillo

**Affiliations:** Department of Neurosciences, Mario Negri Institute for Pharmacological Research IRCCS, Milan, Italy

**Keywords:** myokines, exercise, exerkines, skeletal muscles, atrophy

## Abstract

Skeletal muscle is a highly vascularized tissue that can secrete proteins called myokines. These muscle-secreted factors exert biological functions in muscle itself (autocrine effect) or on short- or long-distant organs (paracrine/endocrine effects) and control processes such as metabolism, angiogenesis, or inflammation. Widely differing diseases ranging from genetic myopathies to cancers are emerging as causing dysregulated secretion of myokines from skeletal muscles. Myokines are also involved in the control of muscle size and may be important to be restored to normal levels to alleviate muscle wasting in various conditions, such as cancer, untreated diabetes, chronic obstructive pulmonary disease, aging, or heart failure. Interestingly, many myokines are induced by exercise (muscle-derived exerkines) and some even by specific types of physical activity, but more studies are needed on this issue. Most exercise-induced myokines travel throughout the body by means of extracellular vesicles. Restoring myokines by physical activity may be added to the list of mechanisms by which exercise exerts preventative or curative effects against a large number of diseases, including the deleterious muscle wasting they may cause. Extending our understanding about which myokines could be usefully restored in certain diseases might help in prescribing more tailored exercise or myokine-based drugs.

## Introduction

Skeletal muscle is the largest tissue in the human body, accounting for about 30% of body mass in women and 40% in men. Although, it can suffer in many incurable diseases, it is unexpectedly the most “undrugged” tissue. Skeletal muscle is very plastic because it can be enlarged with adaptation to specific exercise and/or high-protein content diet or reduced in catabolic conditions like fasting, cancer, untreated diabetes, heart failure, AIDS, chronic obstructive pulmonary disease (COPD), or aging (Piccirillo et al., 2014; Furrer and Handschin, 2018). This tissue is the main body’s protein reservoir, able to break down its own proteins and release aminoacids into the bloodstream during stress or fasting or disease. This process goes under the name of muscle wasting or cachexia, and ultimately aggravates the disease state, even leading individuals to death (Kalantar-Zadeh et al., 2013). Losing more than about 40% of normal body mass was incompatible with life in starved, lager-confined Jews (Winick, 1979) and in HIV-infected patients before the successful advent of antiretroviral therapy (Kotler et al., 1989; Roubenoff and Kehayias, 1991).

Reduction in muscle size mainly consists of a decrease in cell size caused by loss of organelles, cytosol and proteins. This deleterious process is mainly driven by increased protein breakdown through enhanced proteasomal and lysosomal activities (coordinatively activated by FoxO3-dependent transcription) (Sandri et al., 2004; Zhao et al., 2007) and reduced protein synthesis (mainly regulated by the IGF-1/PI3K/AKT pathway). During muscle atrophy, the removal of ATP-producing organelles, such as mitochondria, through enhanced autophagy (i.e., mitophagy) explains the increased propensity to fatigue of muscle-losing patients (VanderVeen et al., 2017).

Physical exercise is among the ways by which muscles may be protected against disease-induced muscle wasting. Physical activity can be grossly divided into two types: aerobic (or endurance) and anaerobic (or strength) exercise. The former shifts muscle fiber types toward those with increased capacity for aerobic metabolism and better ability to resist fatigue, due to a larger number of mitochondria and vessels (types I and IIA fibers), this is typical in runners, long-distance cross-country skiers, bicyclists, swimmers. These fibers contract slowly with a low peak force and generate ATP through oxidative phosphorylation of glucose and non-esterified fatty acids. The second type of exercise causes hypertrophy, especially of myofibers IIX (and IIB in rodents), due to enhanced synthesis of contractile proteins, resulting in increased strength with no change in the number of mitochondria (typically occurring in weight-lifters and body-builders). These fibers generate fast contractions with a high peak force, and metabolize phosphocreatine and glucose anaerobically to make ATP (Flück and Hoppeler, 2003; Hawley et al., 2014).

Aerobic exercise induces in humans (Pilegaard et al., 2003) and in rodents (Terada et al., 2002) the peroxisome proliferator-activated receptor γ coactivator 1-α (PGC1-α). PGC1-α not only promotes mitochondrial biogenesis, contrasting the propensity to fatigue of cachectic muscles, but also directly antagonizes protein catabolism by blocking FoxO3 (Sandri et al., 2006), the master transcription factor coordinating both proteasomal and lysosomal protein degradation (Sandri et al., 2004; Mammucari et al., 2007; Zhao et al., 2007). As a result, oxidative fibers are more resistant to cancer-induced atrophy than glycolytic ones, typically enlarged upon muscle adaptation to anaerobic exercise (Ciciliot et al., 2013). Conversely, anaerobic exercise, by stimulating mainly myofibrillar protein synthesis through activation of the PI3K/AKT pathway and overproduction of IGF-1 (McCall et al., 2003), may obviate muscle wasting by enhancing protein synthesis and inactivating FoxO3 through AKT-mediated phosphorylation (Sandri et al., 2004).

It is becoming clear that physical activity mediates the release from muscles of factors with anti-atrophic effects in an autocrine fashion but, surprisingly, these muscle-derived molecules can even contrast the primary disease, as is the case of the recently identified anti-tumoral molecules of muscle origin (Aoi et al., 2013; Gannon et al., 2015). Some examples are oncostatin M, able to restrain mammary cancer cell growth *in vitro* (Hojman et al., 2011), fatigue substance (F-Substance) isolated from muscles of trained rats and displaying inhibitory effect on the breast cancer cell line MCF-7 (Munoz et al., 2013) and secreted protein acidic and rich in cysteine (SPARC). The latter is induced in plasma of trained individuals or mice and it is able to suppress colon tumorigenesis via regular exercise in mice (Aoi et al., 2013).

Skeletal muscle is a highly vascularized tissue and has secretory abilities (Pedersen and Febbraio, 2008). In fact, muscles release not only aminoacids in response to increased energy demand and fuel the liver to undergo gluconeogenesis, but also proteins to mediate inter-tissue crosstalk. These molecules have been named myokines to underline their muscle origin. The current definition of myokines is “cytokines or peptides which are secreted by skeletal muscle cells and subsequently released into the circulation to exert endocrine or paracrine effects in other cells, tissues or organs” (Pedersen and Febbraio, 2012). Not all myokines are exclusively originating by skeletal muscles. Some myokines are mainly muscle-restricted proteins, like myostatin, while others can also be secreted by other tissues, as is the case of the adipomyokines (for example, IL-8 and MCP-1) (Trayhurn et al., 2011). However, skeletal muscle is probably the main source of most myokines secreted also by other tissues in the circulation because it is amply vascularized and makes up 30–40% of the human body mass.

More than 3000 myokines have been reported (Whitham and Febbraio, 2016) and, among them, it is worth mentioning those identified in humans: angiopoietin-like 4 (ANGPTL4), apelin, brain-derived neurotrophic factor (BDNF), CCL2 or MCP-1, CX3CL1 of fractalkine (FKN), fibroblast growth factor 21 (FGF21), interleukin-6 (IL-6), IL-7, IL-8, IL-15, irisin, leukemia inhibitory factor (LIF), meteorin-like protein (Metrnl), myostatin and SPARC (for a review see Catoire and Kersten, 2015). Myokine secretion is a process conserved among species: *Drosophila melanogaster* (Zhao and Karpac, 2017), adult *Danio rerio* (i.e., Zebrafish) (Rovira et al., 2017), and mammals (Demontis et al., 2013b), among others, all have muscles able to release such factors. The first reported myokine, Interleukin-6 (IL-6), is conserved in *Drosophila melanogaster* where three orthologs exist, Unpaired (Upd) also called Outstretched, Upd2 and Upd3, increases with exercise (Ostrowski et al., 2000) and has anti-inflammatory properties, at least in mammals (Pedersen and Febbraio, 2008). Another example is myostatin that is secreted by skeletal muscles and can stimulate activin type II receptors (ActRII), ultimately leading to muscle atrophy; it is conserved in *Drosophila melanogaster* (myoglianin) and in Zebrafish (myostatin b). Notably, myostatin inactivation by spontaneous missense mutations or intentional genetic ablation results in hypermuscularity in a large number of species (McPherron and Lee, 1997; McPherron et al., 1997; Prontera et al., 2009).

**FIGURE 1:**
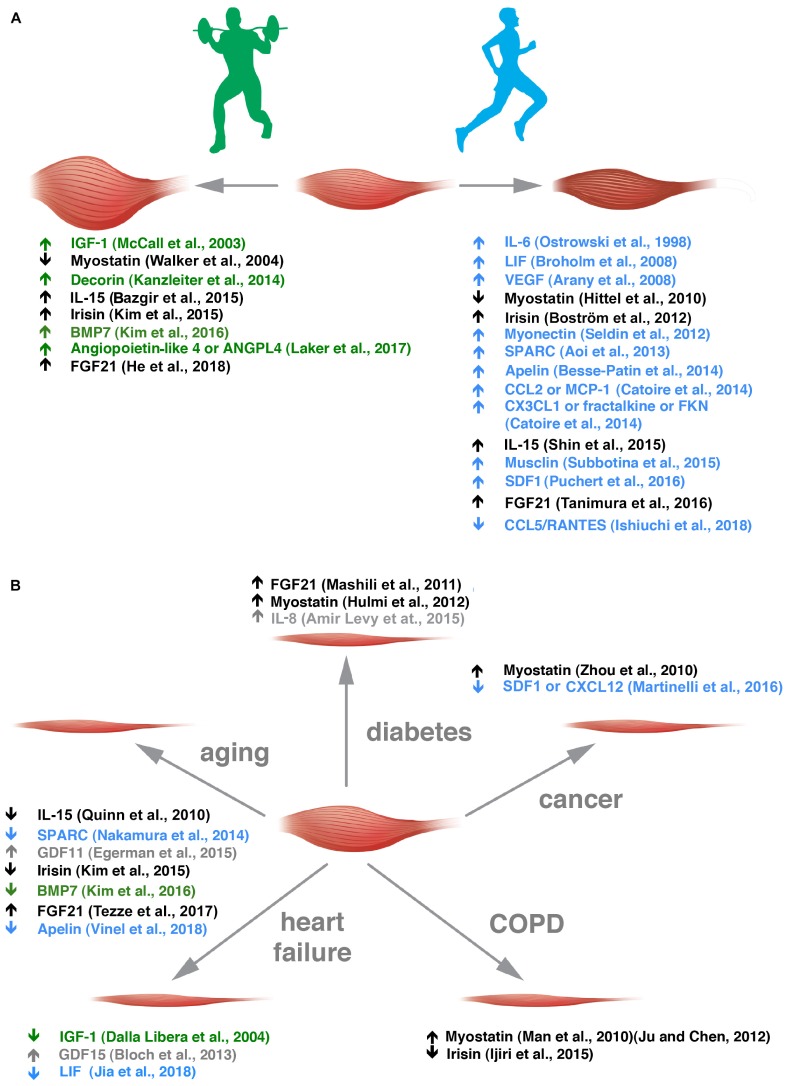
Exercise-sensitive myokines with a preference for aerobic or anaerobic exercise are altered in different muscle wasting disorders. **(A)** Muscle adaptation to anaerobic and aerobic exercise is grossly depicted. In green, myokines altered in anaerobic exercise are listed with the appropriate references. In blue, those altered by endurance training are reported. **(B)** Myokine alterations in the five diseases indicated are listed. Myokines sensitive to anaerobic or aerobic exercise are depicted in green and blue, respectively, and those that change in both kinds of exercise in black. Myokines whose alteration by exercise is not clear are indicated in gray.

Some myokines are preferentially secreted by glycolytic fibers [e.g., musclin (Banzet et al., 2007; Subbotina et al., 2015), osteoprotegerin and angiogenin (Rutti et al., 2018)], and others by oxidative fibers [e.g., myonectin (Seldin et al., 2012)] but the fiber-type preference, if it exists, is unknown for most of the other myokines. Along the same line, FGF21 that is an hormone-like molecule involved in metabolism has been described as an AKT-regulated myokine (Izumiya et al., 2008a; He et al., 2018), hence, more typically secreted by glycolytic fibers, while irisin expression is induced by PGC1α, thus, more typically secreted by oxidative ones (Boström et al., 2012). Some myokines have been renamed muscle-derived exerkines (Safdar and Tarnopolsky, 2018) because they are induced by physical activity and – most interestingly – some of them show a preference for muscle induction through specific types of physical exercise (Figure 1A) (Kanzleiter et al., 2014; Bazgir et al., 2015; Shin et al., 2015; Laker et al., 2017; Ishiuchi et al., 2018).

Lecker et al. (2004) found a subset of genes that were differentially expressed in muscles undergoing wasting in unrelated conditions (fasting, uremia, tumor, and diabetes) in rodent models and named them “atrogenes.” Among them, a subset of potentially secreted proteins, called “extracellular matrix proteins,” were all downregulated in all conditions of muscle atrophy tested and included fibrillin 1, fibronectin 1, galectin 1, osteoblast specific factor 2, secreted modular calcium-binding protein 2, and insulin-like growth factor binding protein 5. It would be worth clarifying whether these include some that could be useful to reverse to basal levels to contrast atrophy, and whether they can be classified as myokines or not (i.e., “atromyokines”).

Most exercise-induced myokines travel through the body by means of exosomes measuring between 30 and 100 nm in diameter, or micro- or nano-sized extracellular vesicles (EVs), through analyses in Vesciclepedia and ExoCarta (Safdar and Tarnopolsky, 2018). These muscle-derived EVs increase in number in the bloodstream upon exercise, perhaps through calcium-dependent secretion coupled with muscle contractions (Whitham et al., 2018). In a very elegant work, Whitham et al. (2018) showed that when EV liberated in the blood of healthy individuals after 1 h bout of cycling exercise were labeled and transferred to donor mice, they were able to target organs like the liver, but not all organs have been scrutinized. The search for exercise factors of muscle origin mediated by these structures has just started and could help explain why myokines are not rapidly degraded by the numerous proteases in the extracellular environment. Their protective lipid bilayers could facilitate them to target muscle-unrelated tissues, affecting overall body homeostasis. This would further help explain how genetic modifications restricted to muscles impact on other tissues (Rai and Demontis, 2016) as the reduced adiposity reported in animals with muscle deficiency of myostatin (McPherron and Lee, 2002) or the increased resistance to diet-induced obesity and steatosis of mice with muscles overexpressing AKT1 (Izumiya et al., 2008b).

The secretion of myokines is altered in a growing number of diseases, including congenital myotonic dystrophy (Nakamori et al., 2017), and modulable through physical activities (Benatti and Pedersen, 2015). This minireview gives an overview of such factors in health and muscle atrophy-causing diseases (Figure 1B) and how physical exercise could correct some of them toward healthier levels, to spare muscle mass.

## Myokines Altered in Diabetes

Tissue cross-talk is emerging as vital to coordinate the different organs implicated in glucose homeostasis. Among the cross-talking factors, muscle-secreted myokines can influence the function and survival of pancreatic β-cells as is the case of angiogenin (previously known as an angiogenesis-promoting factor) (Gao and Xu, 2008) and osteoprotegerin (involved also in bone density’s regulation) (Simonet et al., 1997) that protect β cells from the deleterious effects of proinflammatory cytokines (Rutti et al., 2018).

Diabetes accounts for about 15% of total diseases related to selected myokines (apelin, BDNF, IL-15, irisin, SPARC) (Son et al., 2018). In adult humans, an insulin-resistant state preceding diabetes onset, already alters myokine secretion, as is the case of increased release of myonectin, but not FGF21 or myostatin (Toloza et al., 2018), but the biological effects of this increase are not clear yet. In another study, FGF21 was found increased in the plasma of diabetic individuals, but if this is a compensatory mechanism to increase glucose uptake from muscles remains to be explored (Mashili et al., 2011). This myokine was found increased also in human and murine plasma after a single bout of endurance exercise (Tanimura et al., 2016). In streptozotocin-induced diabetic mice, myostatin is increased and causes muscle wasting (Hulmi et al., 2012). Molecules able to neutralize this myokine are under tests for the treatment of diabetic myopathy in animal models, but with contradictory results so far (Wang et al., 2015; Dong et al., 2016).

Puzzingly, both CX3CL1 (Shah et al., 2011) and Metrln (Chung et al., 2018) were elevated in the blood of patients afflicted by type II diabetes and also in plasma and/or muscles of individuals after acute endurance exercise (Catoire et al., 2014) or short-term interval training (Eaton et al., 2018), but so far their possible contribution to muscle size control has not been explored. Finally, increased plasma apelin has been found to predict type II diabetes but only in men, for reasons we still do not understand (Ma et al., 2014). While the role of apelin in improving insulin resistance by enhancing glucose utilization by muscle and fat is clear (Dray et al., 2008), its direct ability to protect muscles from diabetes-induced atrophy deserves further studies, but its elevated expression upon aerobic exercise is of note (Besse-Patin et al., 2014). IL-8, a chemokine whose muscle secretion is altered by exercise in humans, though mostly after extreme conditions in response to an ultraendurance exercise bout in experienced athletes (Marklund et al., 2013), was found to be overproduced by myotubes from type II diabetes patients and to disfavor muscle capillarization, reducing muscle exposure to glucose, so possibly further impinging on muscle wasting and the primary disease itself (Amir Levy et al., 2015).

Further studies are needed to see whether and which other myokine(s) is/are altered in muscles undergoing diabetic atrophy (for a further summary on this topic see Carson, 2017), and whether it might be useful to target for the cure of this kind of atrophy.

## Myokines Altered in Cancer Cachexia

Muscle wasting occurs in 80% of patients with advanced cancer and causes death in 30–40% of cases (Fearon et al., 2011). Various myokines may be involved in this kind of atrophy (for a review see Manole et al., 2018). Cancer accounts for only 3.3% of total diseases related to selected myokines (apelin, BDNF, IL-15, irisin, SPARC) (Son et al., 2018), but additional myokines have been shown to be more clearly involved in cancer cachexia.

We found stromal derived factor 1 (SDF1), also referred to as CXCL12, as specifically and strongly downregulated only in skeletal muscles of tumor-bearing mice and not in those undergoing wasting for other reasons (i.e., uremia, diabetes, fasting, or disuse) (Martinelli et al., 2016). Overexpression of SDF1 in muscle through *in vivo* electroporation of plasmid spared not only the cross-sectional area of transfected fibers but also that of adjacent untransfected ones, indicating that SDF1 also exerts its anti-atrophic effects in a paracrine fashion (Martinelli et al., 2016). Interestingly, Puchert and coworkers found it to be induced in muscles of mice trained by running for 4 weeks (Puchert et al., 2016). This muscle-secreted factor has a role in myogenesis, muscle regeneration (Bobadilla et al., 2014) and angiogenesis, similarly to VEGF (Arany et al., 2008) and could be involved in increased muscle capillarization upon adaptation to aerobic exercise. Overall, elevated muscle secretion of SDF1 could be ascribed to the mechanisms by which aerobic exercise or some drugs (e.g., Sunitinib) alleviate cancer-associated muscle wasting (Pretto et al., 2015).

Myostatin-based signaling was found increased in muscles of Yoshida hepatoma-bearing rats (Costelli et al., 2008), in C26-bearing mice (Zhou et al., 2010), and in patients with various malignancies (for a review see Han et al., 2013), while in pre-cachectic cancer patients circulating levels of myostatin increased only with certain types of tumors (Aversa et al., 2012). Importantly, while myostatin is the main factor limiting muscle size in mice, it seems that in humans activin A and growth differentiation factor 11 (GDF11) also play major roles and could be targeted to spare muscles during human diseases (Lach-Trifilieff et al., 2014). Anti-ActRII drugs have in fact been tested in cancer patients but unfortunately, there were no improvements in functional parameters (for a review see Furrer and Handschin, 2018). Myostatin is one of the few myokines whose secretion is reduced with both anaerobic (Walker et al., 2004) and aerobic exercise (Hittel et al., 2010), in concordance with its role as a negative modulator of muscle size.

The type of exercise that seems most effective at sparing muscles during cancer seems the aerobic and not the anaerobic one. In fact, the AKT/mTOR signaling appears even hyperactivated in muscles of tumor-bearing rodents (Penna et al., 2010). So, future research should focus on understanding which aerobic exercise-induced myokine(s), besides SDF1, have anticatabolic or proanabolic action in muscles during cancer cachexia.

## Myokines Altered in COPD

Chronic obstructive pulmonary disease is an inexorable lung disease caused by excessive inflammation and subsequent damage to the airways and lung tissue, mainly occurring after long-term exposure to cigarette smoking (Passey et al., 2016).

Very few myokines have been studied in relation to COPD-induced muscle wasting, even though COPD is the most prevalent disease worldwide causing excessive myopenia and death (Lozano et al., 2012). COPD accounts for about 5% of total diseases related to selected myokines (apelin, BDNF, IL-15, irisin, SPARC) (Son et al., 2018). Consistent with its role in reducing muscle mass, myostatin expression is increased in the vastus lateralis (Plant et al., 2010) and quadriceps (Man et al., 2010) from COPD patients. Circulating myostatin protein is also elevated in serum from COPD patients and correlates with reduced muscle mass in males (Ju and Chen, 2012). Clinical trials to test bimagrumab (anti-ActRII-based drug) in COPD patients have recently showed increases in lean body mass and muscle volume in treated patients but unfortunately, no improvements in functional parameters (for a review see, Furrer and Handschin, 2018).

Irisin, a prominent PGC1α-induced myokine, is the cleaved product of the transmembrane protein fibronectin type III domain-containing protein 5 (FNDC5) (Boström et al., 2012) and is induced by resistance exercise in mice and humans (Kim et al., 2015). Most importantly, circulating irisin was low in COPD patients and its levels correlate with exercise capacity (Ijiri et al., 2015). More studies are still needed to dissect the mechanisms behind COPD-related muscle atrophy, because efficacious therapies are lacking.

## Myokines Altered in Aging

Many alterations occur in skeletal muscle with aging (Demontis et al., 2013a), including enhanced activity of the proteasomal pathway causing muscle atrophy (i.e., sarcopenia) (Altun et al., 2010), but the question whether muscle’s ability to secrete myokines changes with aging has only recently started to attract attention. The increased sarcolemmal permeability of aged muscles may favor increased release of myokines during aging. Nonetheless, the expression of bone morphogenetic factor 7 (BMP7) is low in muscles of aged rats but can be restored through either uphill or gradual downhill running (Kim et al., 2016). This myokine may act by stabilizing the neuromuscular junction (NMJ) that is reduced in number and function with aging (for a review see, Demontis et al., 2013b; Larsson et al., 2019). Moreover, BMP7 has been found to cause muscle hypertrophy in mice through Smad1/5-mediated activation of mTOR signaling (Winbanks et al., 2013). Similar results have been obtained for irisin, whose secretion decreases in aging mice and is restored with resistance exercise training (Kim et al., 2015). While myostatin was unchanged in the elderly (Welle et al., 2002), another member of the same transforming growth factor β (TGFβ) family, GDF11, increases in blood (and muscles) during aging in rats and humans and, even more importantly, inhibits muscle regeneration (Egerman et al., 2015). The secretion of FGF21 also seems dysregulated in aged mice (Tezze et al., 2017).

Apelin is an exerkine that has been recently found to have a major role in counteracting age-associated muscle wasting (Vinel et al., 2018), also by promoting mitochondriogenesis, alleviating muscle-related inflammation and stimulating its regenerative capacity. Its possible role in disease-associated muscle atrophy is still unclear. SPARC is induced in plasma of humans by a single bout of steady-state cycling exercise at 70% of VO_2_max for 30 min, or after 4 weeks of cycling, three times a week for 30 min at 70% of VO_2_max, and similarly in exercising mice (Aoi et al., 2013). This protein was low in aged muscle because its internalization in skeletal muscle progenitors becomes defective with age, so its anti-adipogenic effect could be limited and may contribute to fat infiltration in muscle upon aging (Nakamura et al., 2014).

Finally, IL-15 and the soluble form of its receptor also decrease in serum of aged mice (Quinn et al., 2010). These findings are in concordance with the single nucleotide polymorphisms (SNP) found in the human gene for such receptor that affects the muscularity and exercise capacity of individuals (Pistilli et al., 2008) and may influence inter-individual propensity to sarcopenia.

## Myokines Altered in Heart Failure

Muscle wasting in patients with chronic heart failure (HF) identifies those at high risk of death (Anker et al., 1997). IGF-1 decreases in the skeletal muscle of pre-cachectic HF patients (Hambrecht et al., 2002) and in animal models. Treatment with IGF-1 helped contrast the HF-induced muscle atrophy, improving exercise capacity (Dalla Libera et al., 2004).

GDF15 (also known as macrophage-inhibiting factor-1 and involved in appetite regulation) was found elevated in the acute muscle wasting following cardiac surgery and, interestingly, GDF15-treated myotubes undergo wasting, supporting a pro-cachectic role of this factor (Bloch et al., 2013). A recent study showed the effect on muscle expression of an IL-6-like cytokine, LIF, in rats undergoing myocardial infarction. In this model the myocardial infarction-associated muscle wasting was recovered upon interval exercise and LIF was upregulated as well in gastrocnemii (Jia et al., 2018). These results contrast with other reports of a pro-cachectic role of (tumor-derived) LIF in cancer-associated muscle wasting (Seto et al., 2015), so further studies are needed to dissect the signaling pathway activated by LIF in muscle fibers and verify whether endogenous expression of LIF is altered in muscles atrophying because of various conditions. The upregulated expression of LIF by exercise is confirmed by other studies (Broholm et al., 2008) and further proven by the existence in the human LIF promoter of two nuclear factor of activated T cells (NFAT) binding sites (Bamberger et al., 2004), a transcription factor well-known to be activated by muscle contractions through the Ca^2+^ calmodulin-calcineurin axis (Schiaffino and Serrano, 2002).

Searching for similar binding sites in the gene regulatory regions of other putatively exercise-induced myokines should be a topic for future research to expand our knowledge of myokine regulation at the transcriptional level.

## Conclusion

An “exercise pill” to reproduce the multiple beneficial effects of exercise on various organs seems to be unpractical because exercise affects too many interconnected pathways and its holistic effects also depend on the host genetics. Nonetheless, expanding our knowledge about which types of activity in humans control beneficial myokine secretion could give valuable, more personalized suggestions to patients about which kind of exercise to practice. Selected myokine-based drugs could be given to act on specific pathways and help patients who for various reasons cannot exercise.

## Author Contributions

The author confirms being the sole contributor of this work and has approved it for publication. RP conceptualized the manuscript, reviewed the literature, and wrote the manuscript in its entirety.

## Conflict of Interest Statement

The author declares that the research was conducted in the absence of any commercial or financial relationships that could be construed as a potential conflict of interest.
